# Ectopic Kidney Masquerading as a Pelvic Mass: A Case Report

**DOI:** 10.7759/cureus.92050

**Published:** 2025-09-11

**Authors:** Lakshmi Kandhan L, Venkata Ramana K, Velmurugan Palaniyandi, Hariharasudhan Sekar, Sriram Krishnamoorthy

**Affiliations:** 1 Urology, Sri Ramachandra Institute of Higher Education and Research, Chennai, IND

**Keywords:** ectopic kidney, hydronephrosis, nephrectomy, pelvic kidney, pelviureteric junction obstruction, renal anomalies

## Abstract

Renal ectopia is a congenital anomaly caused by abnormal migration of the kidneys during the process of embryogenesis. Under typical development, the kidneys ascend to the lumbar region during the early gestational period. A failure in this process can result in ectopic kidneys, most commonly found in the pelvis. Although often asymptomatic, pelvic kidneys can be complicated by pelviureteric junction obstruction (PUJO), infection, reflux, or malrotation in a few cases. We present a rare case of a pelvic kidney with PUJO presenting as a lower abdominal mass in an elderly female. A 60-year-old female presented to our urology clinic with progressive lower abdominal fullness and vague discomfort for several months. Clinical examination revealed a firm, non-tender mass measuring 15 × 8 cm in the hypogastric and iliac regions. Blood investigations and renal function tests were within normal limits. Contrast-enhanced CT urography revealed a grossly hydronephrotic left pelvic kidney with parenchymal thinning and no contrast excretion. No calculi or masses were identified. A diethylenetriamine pentaacetic acid (DTPA) renogram confirmed severely impaired function of the ectopic kidney. The patient underwent cystoscopy with ureteric catheterization, followed by left nephrectomy. Histopathological examination confirmed chronic pyelonephritis in the hydronephrotic kidney. A pelvic kidney is an uncommon congenital anomaly identified in a small proportion of births. PUJO is one of the most common causes of hydronephrosis in ectopic kidneys. Detailed imaging and functional studies are essential for diagnosis and management. Delayed presentation in adults highlights the need to consider renal ectopia in differential diagnosis. This case emphasizes the importance of recognizing ectopic kidneys in adults presenting with abdominal masses. Timely imaging and functional assessment aid in appropriate surgical decision-making, especially in non-functioning kidneys where nephrectomy prevents long-term complications.

## Introduction

During fetal development, the kidneys ascend to their normal position between the sixth and ninth gestational weeks [1]. When this migration fails, an ectopic kidney results, commonly presenting as a pelvic kidney (1 in 3,000 cases). An ectopic kidney is a congenital anomaly where one or both kidneys fail to ascend to their normal anatomical position in the lumbar retroperitoneal space during embryological development. In such cases, the perirenal fascia is often poorly formed, and the kidney may cross midline. Though modern imaging detects most anomalies early, some ectopic kidneys remain undiagnosed until adulthood, especially in asymptomatic individuals. They may present later with symptoms such as pelviureteric junction obstruction (PUJO) or kidney stones [2]. We report a rare case of an ectopic kidney with PUJO, presenting as an unusually palpable lump in the lower abdomen.

## Case presentation

A 60-year-old female was referred by a general medical practitioner to our outpatient urology clinic with complaints of a progressively enlarging lower abdominal mass and bilateral lower limb edema. The swelling had gradually increased in size over the past four to five years and was occasionally associated with dull, aching pain, which was relieved by over-the-counter analgesics. There was no history of fever, hematuria, urinary tract infections, or other systemic complaints. Patient had no known co-morbidities and no history of previous surgeries.

**Figure 1:**
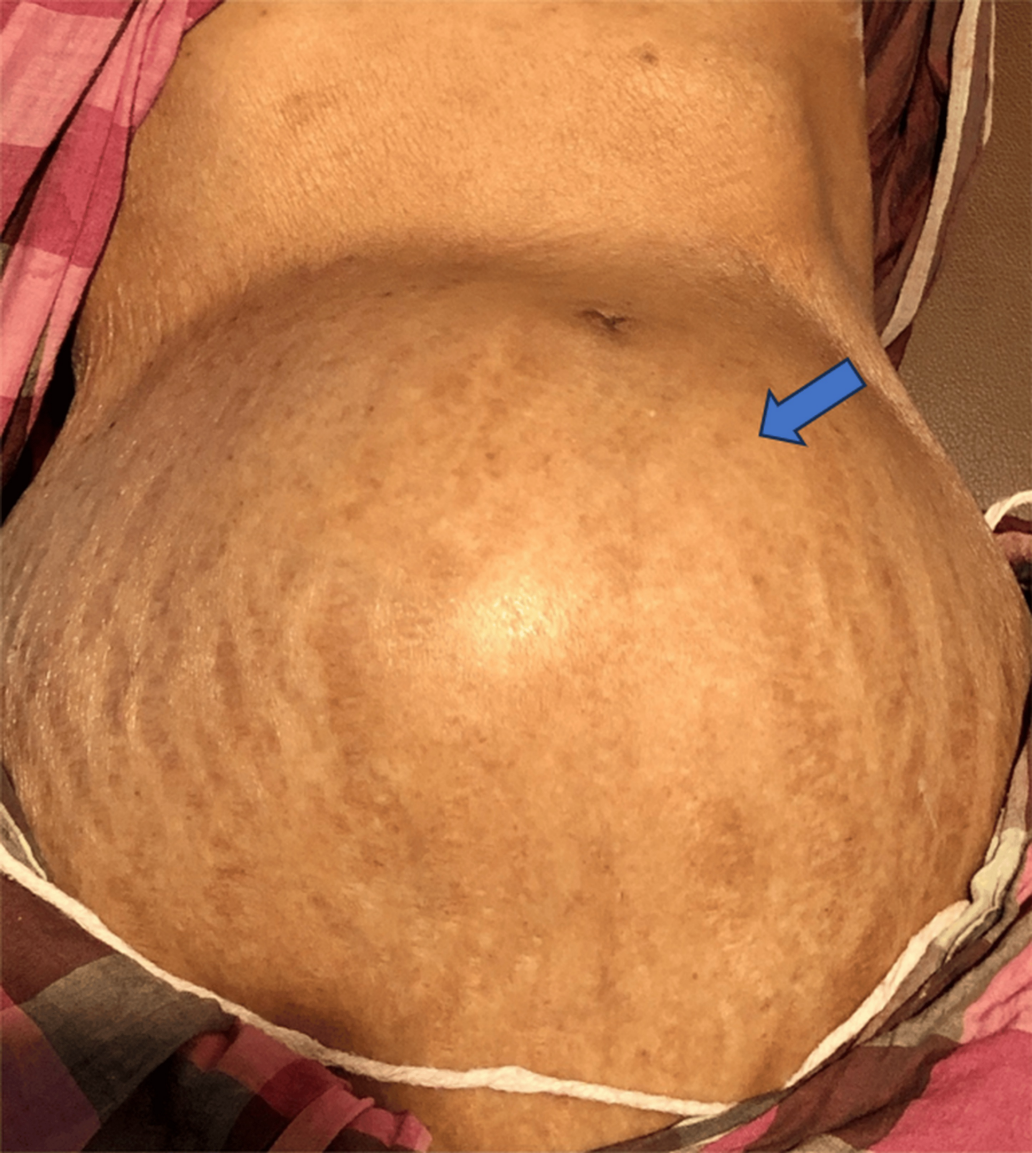
Clinical photograph showing a large lower abdominal mass (blue arrow).

On physical examination, a large, non-tender, cystic mass measuring approximately 15 × 8 cm was palpable in the lower abdomen. The mass extended vertically from 2 cm above the pubic symphysis to the umbilicus and laterally into both iliac fossae. The mass exhibited restricted mobility without signs of inflammation or skin changes (Figure 1).

**Figure 2:**
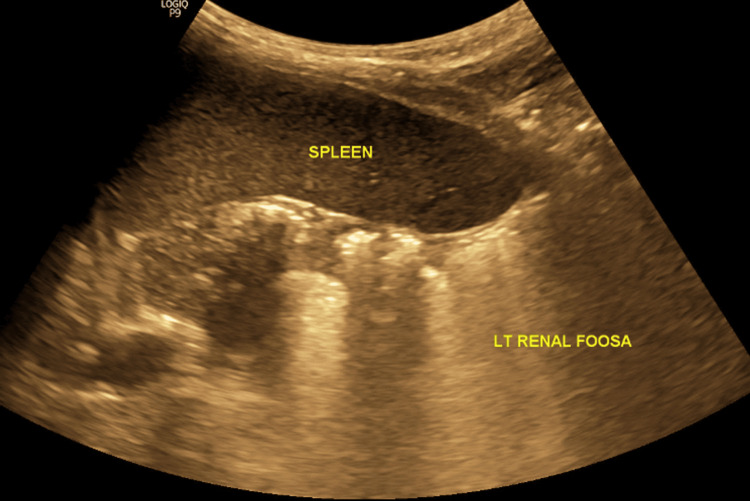
Abdominal ultrasonography showing absence of the left kidney in the renal fossa.

**Figure 3:**
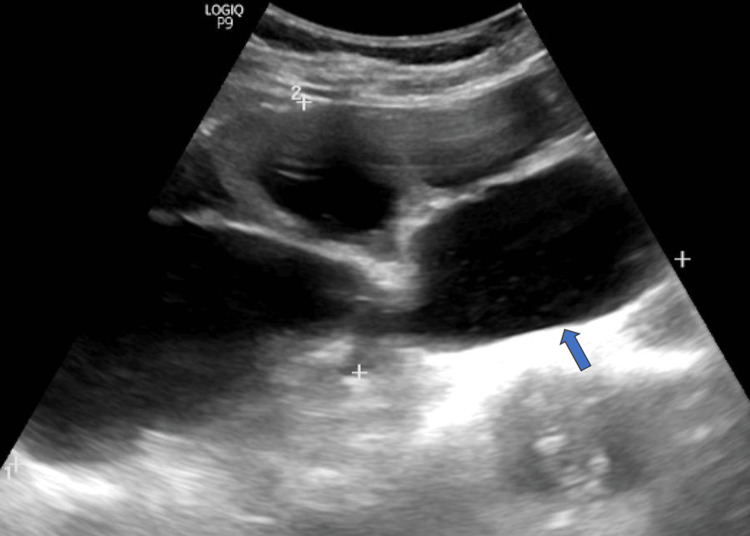
Abdominal ultrasonography showing a large cystic lesion in the pelvic region with no discernible renal cortex (blue arrow), suggestive of a grossly hydronephrotic pelvic kidney.

Blood investigations and renal function tests (serum creatinine: 0.6 mg/dL) were within normal limits. Urine analysis was normal, and urine culture showed no growth. Ultrasonography revealed the absence of the left kidney in the renal fossa (Figure 2) and identified a large cystic lesion in the pelvic region, cranial to the bladder and uterus, with no discernible renal cortex (Figure 3).

**Figure 4:**
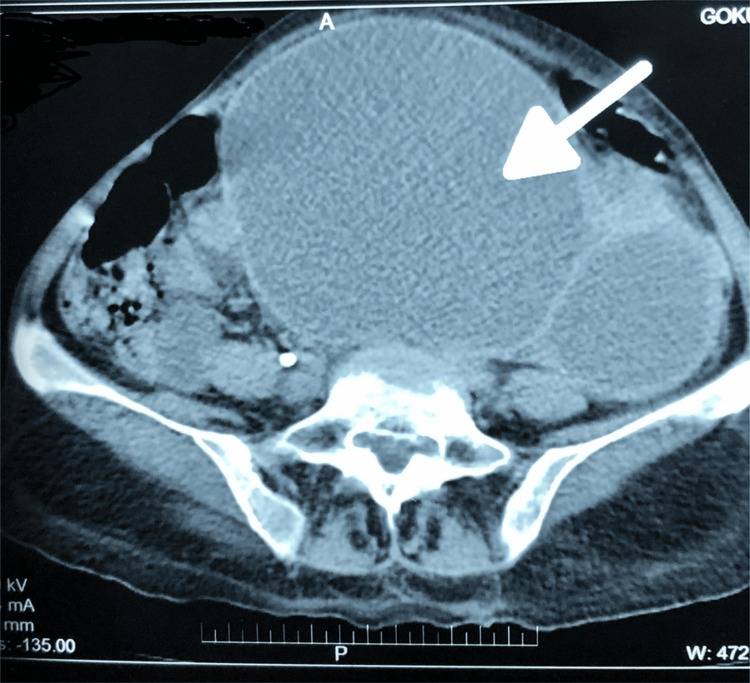
CT scan of the abdomen revealing an ectopic, grossly hydronephrotic left kidney crossing the midline (white arrow).

Contrast-enhanced computed tomography (CT) of the abdomen and pelvis confirmed a grossly hydronephrotic ectopic left kidney located in the left iliac fossa, with severe parenchymal thinning and no contrast excretion even on delayed imaging. The kidney extended across the midline into the contralateral iliac fossa, and the left ureter appeared undilated, suggestive of PUJO (Figure 4).

**Figure 5:**
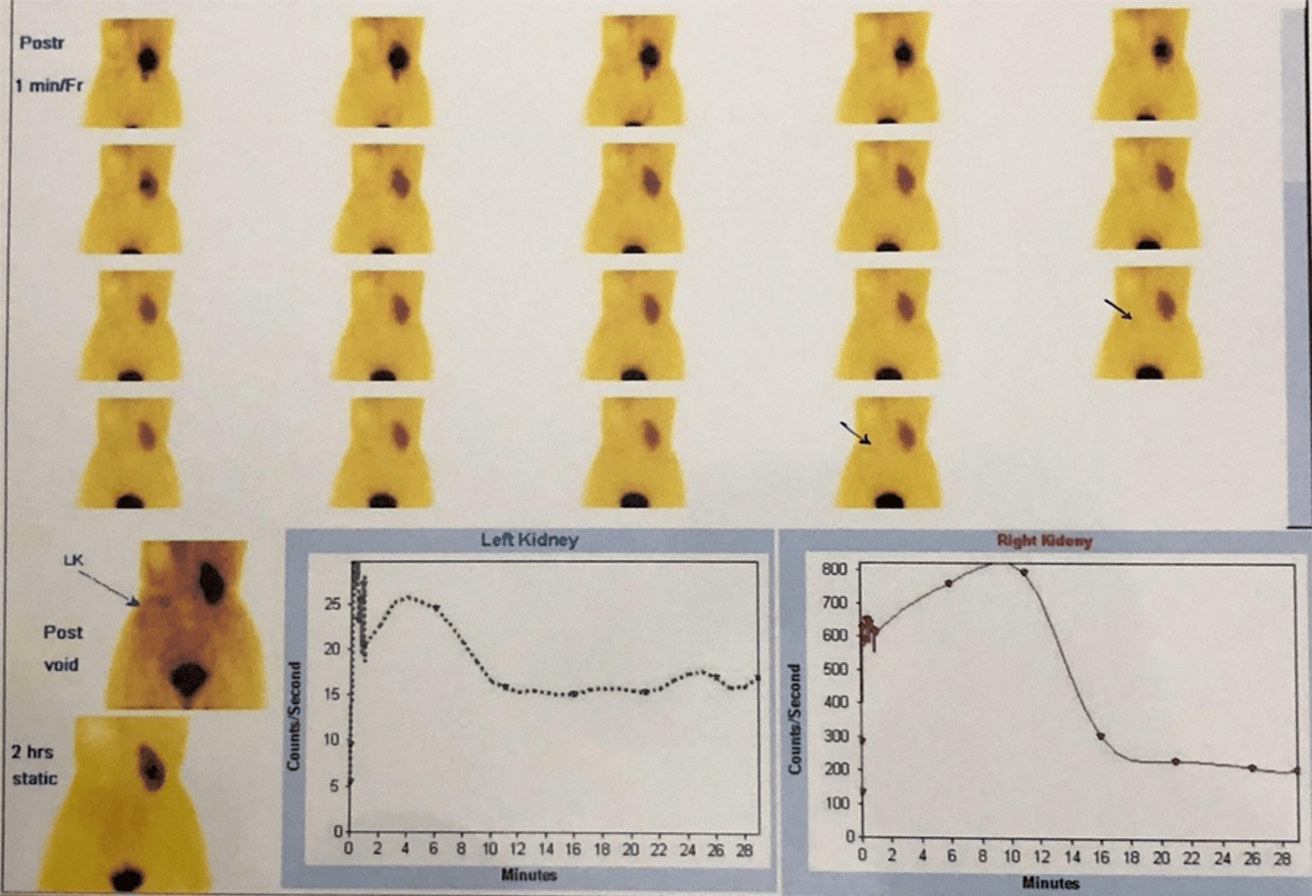
Diethylenetriamine pentaacetic acid (DTPA) renogram indicating a non-functioning left kidney with preserved right kidney function.

A diethylenetriaminepentaacetic acid (DTPA) renogram showed a Type II O’Reilly curve for the left kidney, with a glomerular filtration rate (GFR) of 1.9 mL/min and a relative renal function of 4%. The right kidney demonstrated normal function with a GFR of 54.5 mL/min and a relative function of 96% (Figure 5).

**Figure 6:**
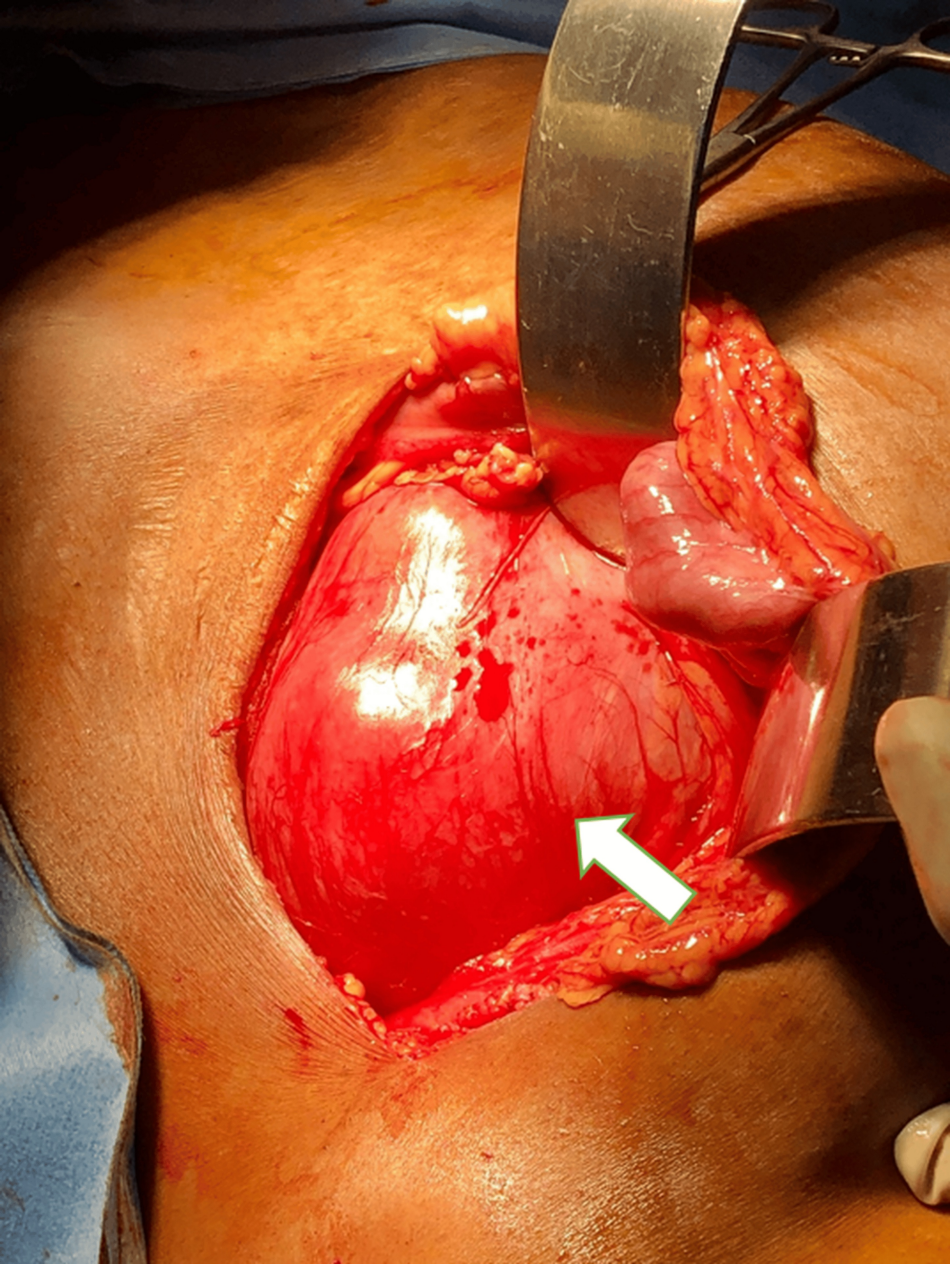
Intraoperative image showing the ectopic left kidney (white arrow).

After completion of the preoperative evaluation, the patient underwent a left open simple nephrectomy by transperitoneal approach via a modified Rutherford Morrison incision. A 5 French (Fr) ureteric catheter was placed retrogradely up to the level of the pelviureteric junction (PUJ) to facilitate intraoperative identification of the ureter. Intraoperatively, a grossly hydronephrotic kidney was noted in the pelvic region without any recognizable Gerota’s fascia (Figure 6).

**Figure 7:**
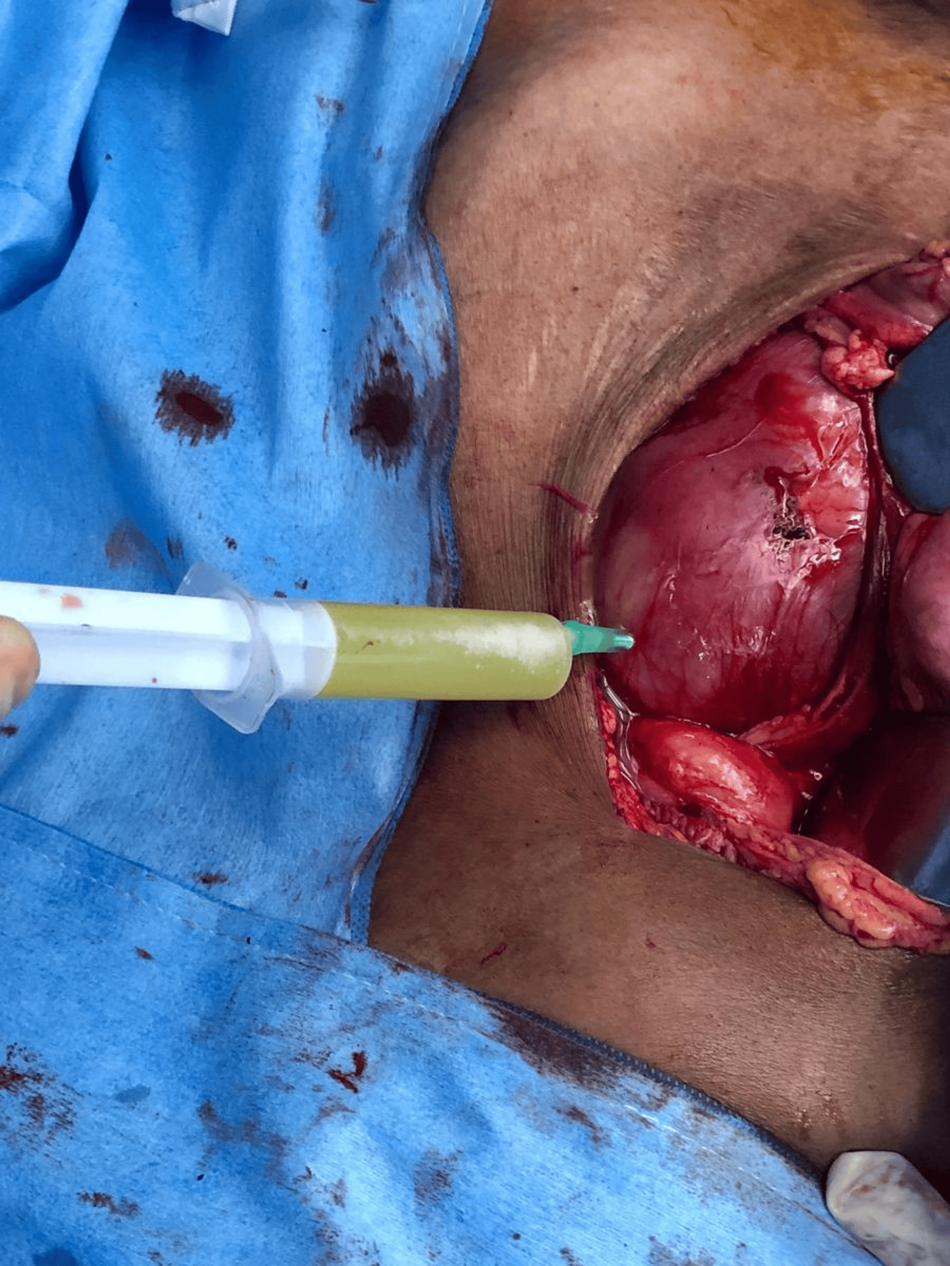
Intraoperative image showing aspiration of turbid urine from the renal pelvis.

Turbid urine was aspirated from the renal pelvis (Figure 7), and a crossing vessel was identified just below the PUJ. Operative time was 90 minutes. The intraoperative period was uneventful, and blood loss was very minimal.

The postoperative period was uneventful. The Foley catheter was removed on postoperative day 3, and the patient was discharged on postoperative day 5 in stable condition. The patient is currently under follow-up and doing well with a right solitary kidney.

## Discussion

Ectopic kidneys, particularly those located in the pelvis, represent a developmental anomaly resulting from failure of normal renal ascent during embryogenesis. These kidneys are often malrotated and can be associated with a spectrum of urological complications, among which hydronephrosis is most commonly observed, affecting up to 50% of such cases [3]. In the present case, the patient harboured a grossly hydronephrotic left pelvic kidney that had crossed the midline and presented as a large, painless abdominal lump - an uncommon but illustrative presentation of longstanding PUJO in an ectopic renal unit.

Hydronephrosis in ectopic kidneys may result from a variety of anatomical and functional causes, including obstruction at the PUJ or vesicoureteric junction (VUJ), vesicoureteric reflux (typically Grade III or higher), or impaired drainage secondary to abnormal renal rotation [4]. The lack of normal rotational development in ectopic kidneys often results in an anteriorly placed renal pelvis. In addition, aberrant vasculature, such as accessory or crossing vessels originating from the iliac arteries or distal aorta, is frequently encountered and may contribute to extrinsic compression at the PUJ, thereby exacerbating obstructive uropathy [5]. Our intraoperative findings corroborated this mechanism, with the identification of a prominent crossing vessel just below the renal pelvis, which likely played a role in the progressive hydronephrosis observed.

The anatomical positioning of pelvic kidneys further complicates the clinical scenario. In their normal lumbar location, kidneys are enveloped by Gerota’s fascia - a fibrous sheath that provides structural support and anatomical compartmentalization. This fascia is fused medially, laterally, and superiorly, while remaining open inferiorly, where it merges with the extraperitoneal connective tissue of the iliac fossa [6]. In contrast, pelvic kidneys are generally devoid of this fascial covering. This absence may reduce the mechanical containment of the kidney, allowing for exaggerated dilation of the renal pelvis and even midline extension, as seen in our patient [7].

Despite such predispositions, many patients with ectopic kidneys remain asymptomatic for extended periods. Obstruction may be partially compensated or progress insidiously, delaying clinical manifestation [8]. In our case, the patient's presentation in the sixth decade of life with a longstanding abdominal mass and no acute urological symptoms highlights the silent nature of this pathology. It underscores the importance of maintaining a high index of suspicion for renal ectopy in patients presenting with lower abdominal swellings, particularly when routine ultrasound fails to demonstrate a kidney in its expected anatomical location.

Imaging plays a pivotal role in diagnosis and treatment planning. The absence of the kidney in the renal fossa on ultrasonography should prompt further evaluation for ectopic locations. Cross-sectional imaging with contrast-enhanced CT not only confirms the anatomical site and structural pathology but also delineates vascular anatomy - information crucial for surgical decision-making. Functional assessment with radionuclide renography, such as DTPA scan, is indispensable in quantifying renal contribution and determining the viability of reconstructive intervention [9]. In our patient, the ectopic kidney demonstrated severely compromised function (GFR: 1.9 mL/min; relative function: 4%) and a non-excreting drainage pattern, indicating a poor prognosis for salvage.

Management strategies for ectopic kidneys with PUJO must be individualized. In kidneys with preserved parenchyma and functional reserve, pyeloplasty - either open, laparoscopic, or robotic - can provide definitive relief and protect renal function [10]. However, in cases such as ours, where function is nearly lost and the kidney has become a potential nidus for infection and secondary complications, nephrectomy is the treatment of choice. Surgical removal eliminates the risks of pyelonephritis, nephrolithiasis, chronic pain, and secondary hypertension, all of which are documented sequelae of poorly functioning obstructed kidneys [11].

This case exemplifies the diagnostic challenges and therapeutic considerations in managing ectopic pelvic kidneys with PUJO. It underscores the importance of comprehensive anatomical and functional assessments. It supports the utility of nephrectomy in cases with non-functioning renal units, particularly when anatomical distortion and chronicity have precluded timely intervention.

## Conclusions

While PUJO is often identified antenatally with modern imaging, ectopic kidneys may remain undiagnosed until adulthood, presenting with symptoms or as incidental findings. This case highlights the importance of considering renal ectopy in atypical abdominal masses. CT and diuretic renography are essential for anatomical and functional assessment. Management should be individualized. Salvageable units may benefit from pyeloplasty, whereas non-functioning kidneys warrant nephrectomy to prevent complications.
